# Pilot Case Series of Lateral Ridge Augmentation Using a Collagenated Porcine-Derived Xenograft: Clinical, Histological, and Remodeling Outcomes

**DOI:** 10.3390/jcm15114171

**Published:** 2026-05-28

**Authors:** Alexandru Spînu, Felicia Manole, Claudia Florina Bogdan-Andreescu, Cristina-Crenguţa Albu, Lavinia-Florica Mărcuț, Roxana Daniela Brata, Alexia Manole, Alexandru Burcea

**Affiliations:** 1 Doctoral School, Faculty of Medicine and Pharmacy, University of Oradea, 410068 Oradea, Romania; spinu.alexandru@didactic.uoradea.ro; 2 Spinu Dental Clinic, Spinu Learning, 410155 Oradea, Romania; 3 Surgical Disciplines Department, Faculty of Medicine and Pharmacy, University of Oradea, 410068 Oradea, Romania; fmanole@uoradea.ro (F.M.); lmarcut@uoradea.ro (L.-F.M.); 4 Department of Speciality Disciplines, “Titu Maiorescu” University, 031593 Bucharest, Romania; alexandru.burcea@prof.utm.ro; 5 Department of Genetics, Faculty of Dentistry, “Carol Davila” University of Medicine and Pharmacy, 020021 Bucharest, Romania; 6 Medical Disciplines Department, Faculty of Medicine and Pharmacy, University of Oradea, 410068 Oradea, Romania; brata.roxanadaniela@didactic.uoradea.ro; 7 Faculty of Medicine and Pharmacy, University of Oradea, 410068 Oradea, Romania; manole.alexia@student.uoradea.ro

**Keywords:** biomaterial integration, foreign body reaction, histomorphometry, osteoclastic activity, osteoconduction, peri-implant regeneration, scaffold remodeling

## Abstract

**Background/Objectives:** Horizontal alveolar ridge resorption following tooth loss often compromises implant placement and requires augmentation procedures to restore adequate bone volume. This pilot case series descriptively evaluated the clinical, radiographic, and histological outcomes of lateral ridge augmentation (LRA) using a collagenated porcine-derived xenograft combined with autogenous bone. **Methods:** Three consecutive partially edentulous patients presenting with severe horizontal ridge deficiency (residual bone width ≤ 4 mm) underwent LRA using a mixture of porcine-derived xenograft and autogenous bone covered with a resorbable collagen membrane. After a healing period of 3–5 months, core biopsies were harvested at implant placement and subjected to histological and histomorphometric analysis, including TRAP staining. **Results:** All sites healed uneventfully without intraoperative or postoperative complications. Radiographic evaluation demonstrated substantial horizontal bone gain, allowing placement of standard-diameter implants. Histological analysis revealed newly formed trabecular bone, residual graft material, and well-vascularized connective tissue, indicating active bone regeneration and biomaterial integration. TRAP-positive multinucleated giant cells (MNGCs) were observed at the biomaterial interface, suggesting ongoing remodeling. Long-term follow-up (mean 54.2 months) showed stable implant function without biological or mechanical complications. **Conclusions:** Within the limitations of this pilot case series, LRA using a collagenated porcine-derived xenograft combined with autogenous bone demonstrated preliminary favorable clinical, radiographic, and histological outcomes.

## 1. Introduction

Alveolar ridge resorption following tooth loss is a well-documented biological process characterized by progressive horizontal and vertical bone loss, which may compromise optimal implant placement and prosthetic outcomes [1,2]. The extent of resorption is influenced by time, anatomical location, and local and systemic factors, with more pronounced dimensional changes observed in long-standing edentulous areas and in the presence of iatrogenic or inflammatory conditions [3,4]. As a consequence, insufficient ridge width frequently necessitates augmentation procedures prior to implant placement.

Guided bone regeneration (GBR) has become a widely accepted and predictable technique for the reconstruction of deficient alveolar ridges, with numerous studies demonstrating favorable clinical outcomes and high implant survival rates in regenerated bone [5,6]. Among the various augmentation approaches, lateral ridge augmentation (LRA) is particularly indicated for the correction of horizontal defects. However, it remains a technique-sensitive procedure, often associated with increased risk of complications and variable long-term dimensional stability when compared with other augmentation techniques, such as sinus floor elevation [7,8,9,10].

Several GBR strategies have been proposed for LRA, including the use of particulate grafts in combination with resorbable or non-resorbable membranes, titanium-reinforced barriers, and composite grafts combining autogenous bone with xenogenic or allogeneic materials [11,12,13,14]. The success of these approaches depends on key biological principles, including space maintenance, graft stabilization, vascularization, and exclusion of soft tissue infiltration [15,16]. Despite the wide range of available techniques, clinical outcomes remain influenced by defect morphology, anatomical site, and the biological behavior of the grafting material [17,18].

Autogenous bone has long been regarded as the gold standard because of its osteogenic, osteoinductive, and osteoconductive capabilities. Nevertheless, its clinical use is constrained by donor-site morbidity, greater surgical complexity, and limited supply [19]. As a result, different biomaterials, including allografts and xenografts, have become increasingly utilized in clinical practice.

Xenogenic bone substitutes, particularly those derived from bovine sources, have demonstrated long-term clinical success due to their excellent osteoconductive properties and volumetric stability [20,21]. Nevertheless, their slow resorption rate may result in long-term persistence of residual graft material, raising concerns regarding complete remodeling and replacement by vital bone [22]. In this context, porcine-derived xenografts have been introduced as an alternative, aiming to achieve a more favorable balance between scaffold stability and physiological resorption [23,24]. Furthermore, collagenated porcine-derived biomaterials may improve handling characteristics and enhance cellular colonization by preserving native collagen components [25].

Beyond improved handling characteristics, collagenated porcine-derived xenografts may also exhibit distinct biological behavior in comparison with non-collagenated xenografts. Preservation of native collagen components may support early cellular adhesion, vascular infiltration, and scaffold colonization, potentially supporting more favorable integration and remodeling within the host tissue [26,27,28]. In contrast, non-collagenated xenografts primarily serve as mineral scaffolds with slower biological interactions, often prioritizing long-term structural stability over early regenerative dynamics [29]. Therefore, collagenated formulations may offer a biologically advantageous balance between scaffold stability and physiological remodeling, although comparative human histological evidence remains limited.

Despite the increasing clinical use of porcine-derived xenografts, their biological behavior in lateral ridge augmentation remains insufficiently documented, particularly in human studies. Histological and histomorphometric analyses represent the gold standard for evaluating bone regeneration, allowing quantitative assessment of newly formed bone, residual graft material, and connective tissue. In addition, histochemical techniques such as tartrate-resistant acid phosphatase (TRAP) staining enable the identification of osteoclastic activity, providing further insight into biomaterial resorption and remodeling dynamics at the graft–bone interface.

To date, only a limited number of clinical studies have combined histomorphometric evaluation with TRAP staining in human subjects, especially in the context of LRA using collagenated porcine-derived xenografts. Therefore, this pilot case series aimed to provide preliminary descriptive clinical, radiographic, histological, histomorphometric, and TRAP-based observations regarding the healing and remodeling characteristics of a collagenated porcine-derived xenograft combined with autogenous bone in human lateral ridge augmentation sites. Particular emphasis was placed on documenting early bone formation, graft integration, and biomaterial-associated remodeling processes under clinical conditions where human histological evidence remains limited.

## 2. Materials and Methods

### 2.1. Study Design and Ethical Approval

This retrospective pilot case series included three patients who required implant-supported rehabilitation with lateral bone augmentation in the maxilla or mandible due to insufficient residual bone width (RBW). All surgical procedures were carried out as part of routine clinical care between September 2021 and January 2022. Ethical approval was subsequently obtained in December 2024, specifically for the retrospective analysis and publication of anonymized clinical, radiographic, and histological data.

The study was conducted in accordance with the principles of the Declaration of Helsinki and received ethical approval for the retrospective analysis and publication of anonymized data from the Bioethics Committee of Dr. Spinu Dental Clinic, Oradea (Approval No. 02/12.12.2024).

All patients provided written informed consent for the surgical procedures and for the use of anonymized clinical, radiological, and histological data for research and publication purposes.

This retrospective pilot case series was reported primarily in accordance with the CARE (CAse REport) guidelines for structured case series presentation, while also incorporating relevant STROBE (Strengthening the Reporting of Observational Studies in Epidemiology) principles applicable to observational retrospective analyses, particularly regarding patient selection transparency, eligibility criteria, bias acknowledgment, and outcome reporting.

### 2.2. Patient Selection

This case series included three patients who underwent lateral bone augmentation with collagenated porcine xenograft mixed with autogenous bone and staged implant placement during the study period, and in whom bone biopsies could be obtained ethically.

**Figure 1:**
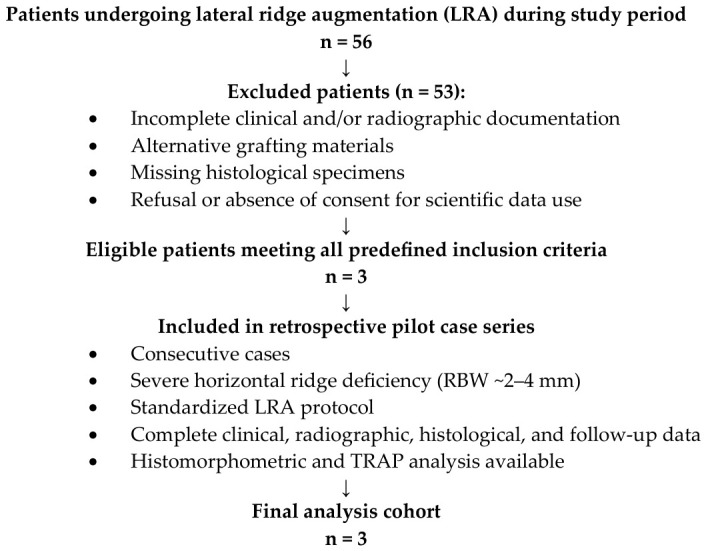
Patient Selection Flowchart.

Between 2 September 2021 and 31 January 2022, 56 patients underwent lateral ridge augmentation procedures at our clinic. Only patients fulfilling all of the following criteria were included in this retrospective pilot analysis: severe horizontal ridge deficiency requiring standardized staged LRA, availability of complete clinical and radiographic documentation, ethically obtained bone core biopsies at implant placement, and complete histological processing. The requirement for biopsy availability substantially limited the eligible cohort. Consequently, only three consecutive patients fulfilled all predefined criteria and were included (Figure 1). This highly selective inclusion process may introduce selection bias and limit generalizability.

Eligibility criteria for inclusion in this retrospective analysis were: severely atrophic maxilla or mandible with residual bone width (RBW) approximately 2 mm, requiring lateral bone augmentation, and availability of complete clinical, radiological, and histological documentation.

All included patients were in good general health (American Society of Anesthesiologists (ASA) Physical Status I). GBR was performed using a collagenated porcine-derived xenograft in combination with autogenous bone and resorbable membrane.

During the second surgery for implant placement, a bone core biopsy was collected to assess graft integration. All patients had complete clinical and radiographic records and provided written informed consent for the use of anonymized clinical, radiological, photographic, and histological data for research and publication purposes.

Exclusion criteria included smoking, systemic conditions that could influence bone healing, incomplete case documentation, use of alternative grafting materials, or refusal to provide informed consent.

**Figure 2:**
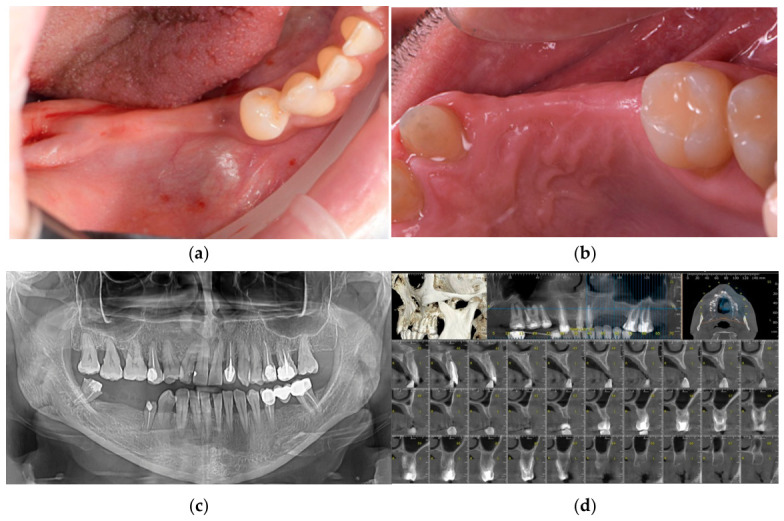
Baseline clinical and radiographic characteristics prior to treatment. (**a**) Clinical preoperative view of a narrow mandibular alveolar ridge with marked horizontal bone deficiency in the posterior region, indicating the need for LRA prior to implant placement; (**b**) Clinical preoperative view of a horizontally atrophic maxillary alveolar ridge in the anterior region, showing significant reduction in ridge width and confirming the indication for LRA; (**c**) Preoperative OPG obtained at presentation, prior to tooth extraction, illustrating the dental status and baseline alveolar bone conditions; (**d**) Preoperative CBCT scan demonstrating horizontal alveolar ridge deficiency with reduced bone width at the planned implant site.

Three patients fulfilled all inclusion criteria and were enrolled in this pilot study. The study population consisted of partially edentulous individuals (one male and two females; age range: 34–62 years) seeking oral rehabilitation to restore oral functionality (Figure 2a,b).

Preoperative assessment included orthopantomography (OPG) and cone-beam computed tomography (CBCT) for all patients to evaluate the defect morphology and plan the surgical procedure (Figure 2c,d).

Based on CBCT findings and patient-specific considerations, the treatment plan consisted of lateral ridge augmentation with staged implant placement, followed by prosthetic rehabilitation after approximately 6 months of healing.

All cases were managed according to a standardized staged treatment protocol to ensure consistency in clinical procedures and histological assessment. Likewise, all histological specimens were processed using a standardized protocol to provide consistent evaluation of new bone formation and graft remodeling.

A staged treatment protocol was adopted, including GBR, delayed implant placement with soft tissue augmentation, followed by vestibuloplasty with connective tissue graft and subsequent prosthetic rehabilitation.

The following treatment protocol was applied in this case series for lateral ridge augmentation of narrow alveolar ridges:

- GBR was performed in healed ridges at least 2 months after tooth extraction, using a particulate collagenated porcine-derived xenograft combined with autogenous bone;
- Following a healing period of 3–5 months, implant placement was carried out, with connective tissue grafting performed when indicated;
- In posterior sites, no temporary prosthetic restoration was used, whereas in anterior regions, a removable interim prosthesis (Snap-On Smile^®^ type (DenMat Lab, Lompoc, CA, USA)) was provided to avoid pressure on the grafted area;
- A closed connective tissue graft was performed approximately 3 months later to improve peri-implant soft tissue conditions;
- Implant uncovering and final prosthetic rehabilitation were completed after an additional 3 months.

### 2.3. Surgical Procedure

#### 2.3.1. Preoperative Preparation

According to the institutional clinical protocol, perioperative antibiotic prophylaxis consisted of amoxicillin/clavulanic acid 875/125 mg (Augmentin^®^, GlaxoSmithKline, London, UK), prescribed twice daily. Treatment was initiated 24 h prior to surgery and maintained for 5–7 days following the procedure.

The use of antibiotics was based on the surgeon’s clinical judgment, considering the extent of the surgical procedure, the use of biomaterials, and the potential risk of postoperative infection. This regimen reflected the institutional clinical protocol applied during the study period for extensive regenerative procedures involving biomaterials and staged reconstruction. Antibiotic selection and duration were based on surgical judgment, considering procedural complexity and perceived infection risk. All patients included in this case series were systemically healthy and did not present conditions requiring therapeutic antibiotic administration.

Immediately prior to surgery, patients performed a 0.2% chlorhexidine mouth rinse to reduce the intraoral bacterial load. Local anesthesia was administered using 4% articaine hydrochloride with epinephrine 1:100,000 (Ubistesin forte^®^, 3M ESPE, Seefeld, Germany) to ensure adequate surgical analgesia.

#### 2.3.2. Lateral Ridge Augmentation

**Figure 3:**
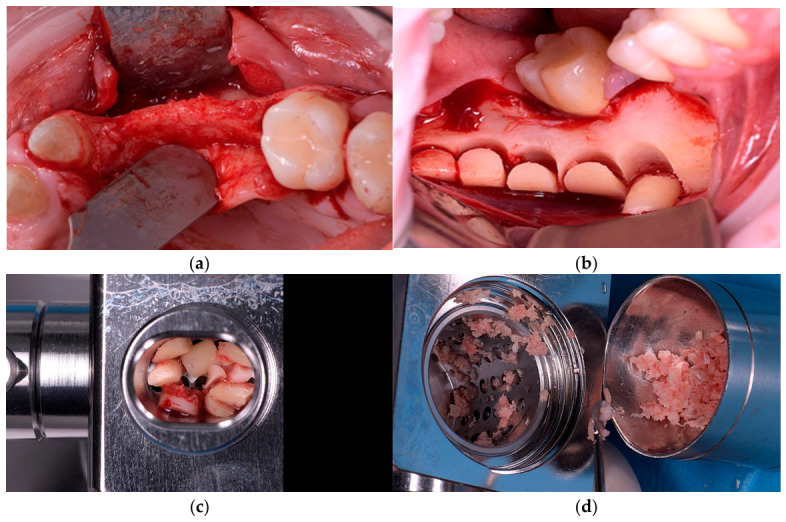

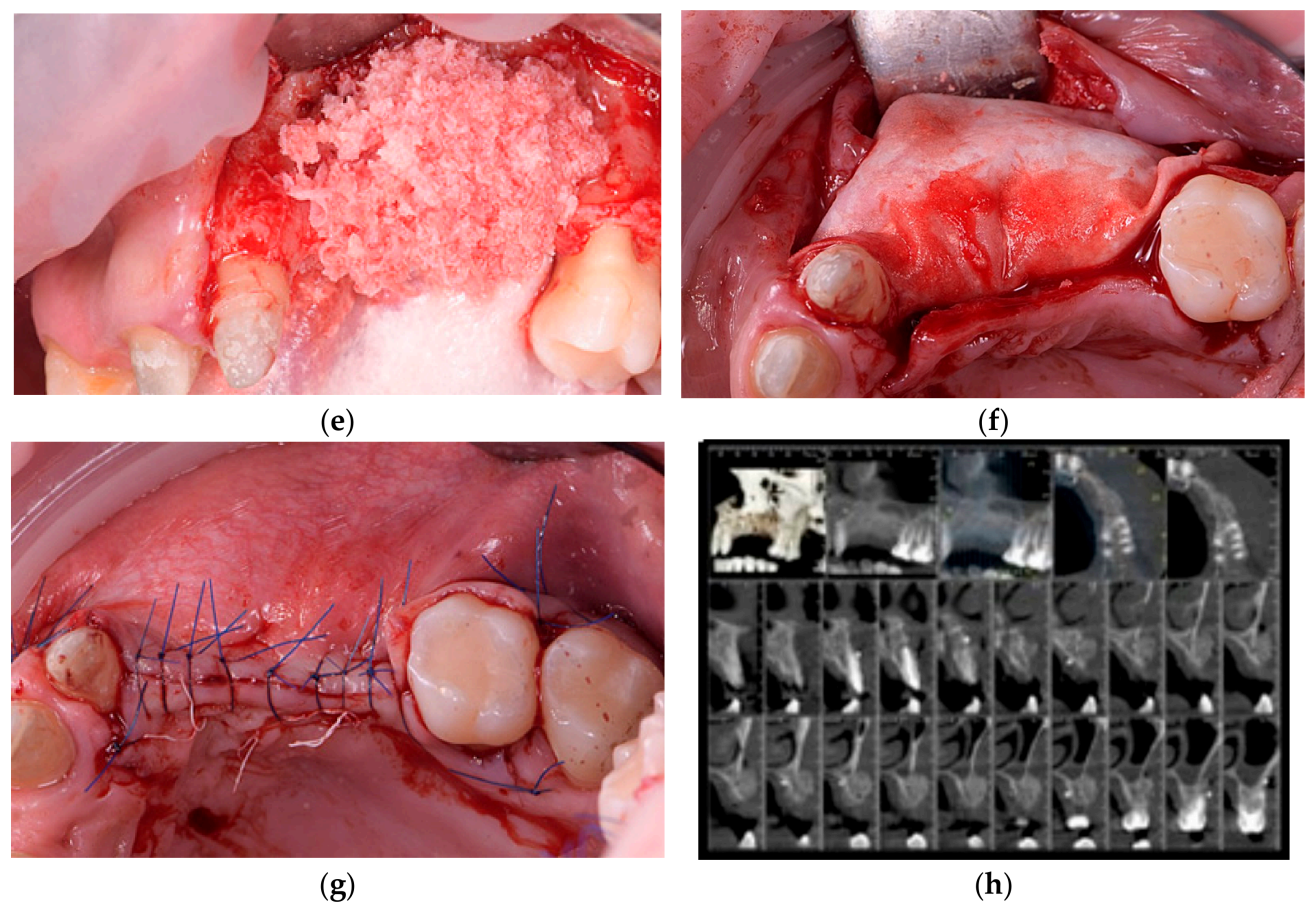
Representative intraoperative and postoperative stages of LRA. (**a**) Recipient site exposure with a RBW of approximately 2 mm; (**b**–**d**) Autogenous bone harvesting and particulation, (**e**) Graft placement; (**f**) Membrane stabilization; (**g**) Flap closure; (**h**) Postoperative CBCT scan showing the augmented ridge volume.

A crestal incision combined with vertical releasing incisions was used to obtain adequate surgical access, followed by elevation of a full-thickness mucoperiosteal flap (Figure 3a). Vertical incisions were positioned anteriorly and distally, one to two teeth away from the ridge defect; in edentulous areas, they were placed at least 5 mm beyond the planned augmentation margins to help preserve vascular supply.

Selective cortical perforations were then created at the recipient site using a small round bur to stimulate bleeding, support the migration of marrow-derived cells, and help with vascularization.

Small autogenous bone blocks were harvested from the external oblique line of the mandible using a 6 mm trephine bur (Helmut Zepf, Seitingen-Oberflacht, Germany) and subsequently particulated using a bone mill (Meisinger, Neuss, Germany) (Figure 3b–d). Autogenous bone harvesting from the external oblique ridge was limited to approximately 1–2 cc per case, corresponding to the volume required for standardized graft mixing. This minimally invasive harvesting approach was selected to reduce donor-site morbidity while providing sufficient autogenous material for composite grafting. The particulated autogenous bone was mixed with a small-particle collagenated porcine-derived xenograft (THE Graft™, Purgo Biologics, Gyeonggi-do, Republic of Korea) in a 1:1 ratio and placed onto the recipient site with slight overcontouring to compensate for potential resorption (Figure 3e). A standardized 1:1 ratio of autogenous bone and xenogenic graft material was selected based on established GBR principles, aiming to combine the biological activity of autogenous bone with the volumetric stability and scaffold properties of xenogenic biomaterials [30,31,32].

Following graft placement, the augmented site was covered with a resorbable collagen membrane (THE Cover™, Purgo Biologics Inc., Seongnam-si, Gyeonggi-do, Republic of Korea) and stabilized using 6–8 fixation pins to maintain space and immobilize the graft material (Figure 3f). Pin fixation was performed at the mesio-lingual, disto-lingual, disto-buccal, mesio-buccal, middle-lingual, and middle-buccal aspects in accordance with the technique described by Urban [7]. The membrane completely covered the grafted area and extended beyond the defect boundaries. On the oral aspect, additional stabilization was achieved using 6-0 polyglycolidecaprolactone (PGCL) monofilament resorbable sutures (Monofast^®^, Assut Europe S.p.A., Pescara, Italy).

A periosteal releasing incision was made on the inner aspect of the flap, as close as possible to its base, extending through the periosteum into the mucosal layer to facilitate tension-free flap advancement and primary closure. The flap was subsequently mobilized, repositioned, and sutured without tension using 4-0 PTFE sutures (Biotex™, Purgo Biologics Inc., Seongnam-si, Gyeonggi-do, Republic of Korea) (Figure 3g). An immediate postoperative CBCT scan was obtained to verify graft position and augmented volume (Figure 3h).

Postoperative management included nimesulide 100 mg (Aulin^®^, Angelini Pharma, Bucharest, Romania), administered every 12 h for pain and inflammation control. The preoperative antibiotic regimen of amoxicillin/clavulanic acid 875/125 mg was continued for 7 days after surgery in accordance with the institutional protocol.

Patients were advised to apply cold packs during the first 48 h, follow a soft diet until suture removal, and avoid sleeping on the operated side. Sutures were removed after 10–14 days.

Postoperative oral hygiene was maintained with saline rinses while mechanical brushing at the surgical site was avoided. Patients were also instructed to refrain from strenuous physical activity for 2–4 weeks to support graft stability.

Mild discomfort and swelling were considered within normal postoperative limits. No postoperative infections, adverse events, or membrane exposure were recorded. No donor-site complications, including infection, excessive pain, sensory disturbances, or delayed healing, were observed. Postoperative morbidity was clinically comparable to that typically associated with routine mandibular third molar extraction.

Patients were advised to promptly report any persistent bleeding, escalating pain, or other unusual postoperative symptoms.

#### 2.3.3. Implant Placement and Biopsy Harvesting

**Figure 4:**
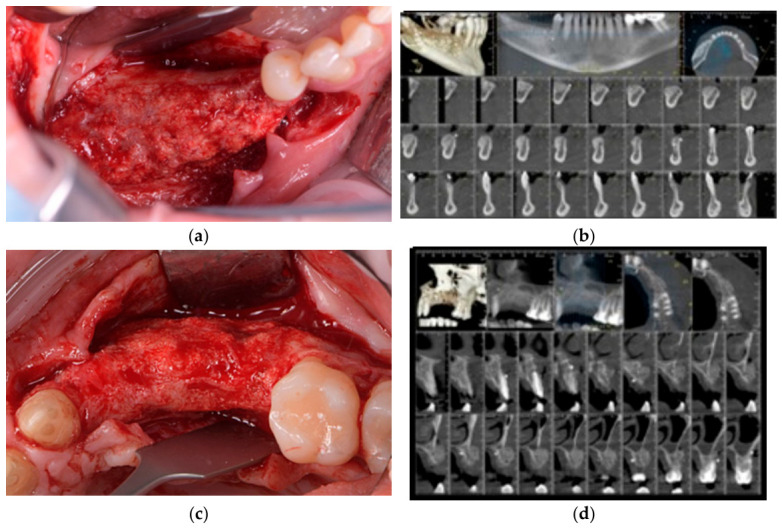

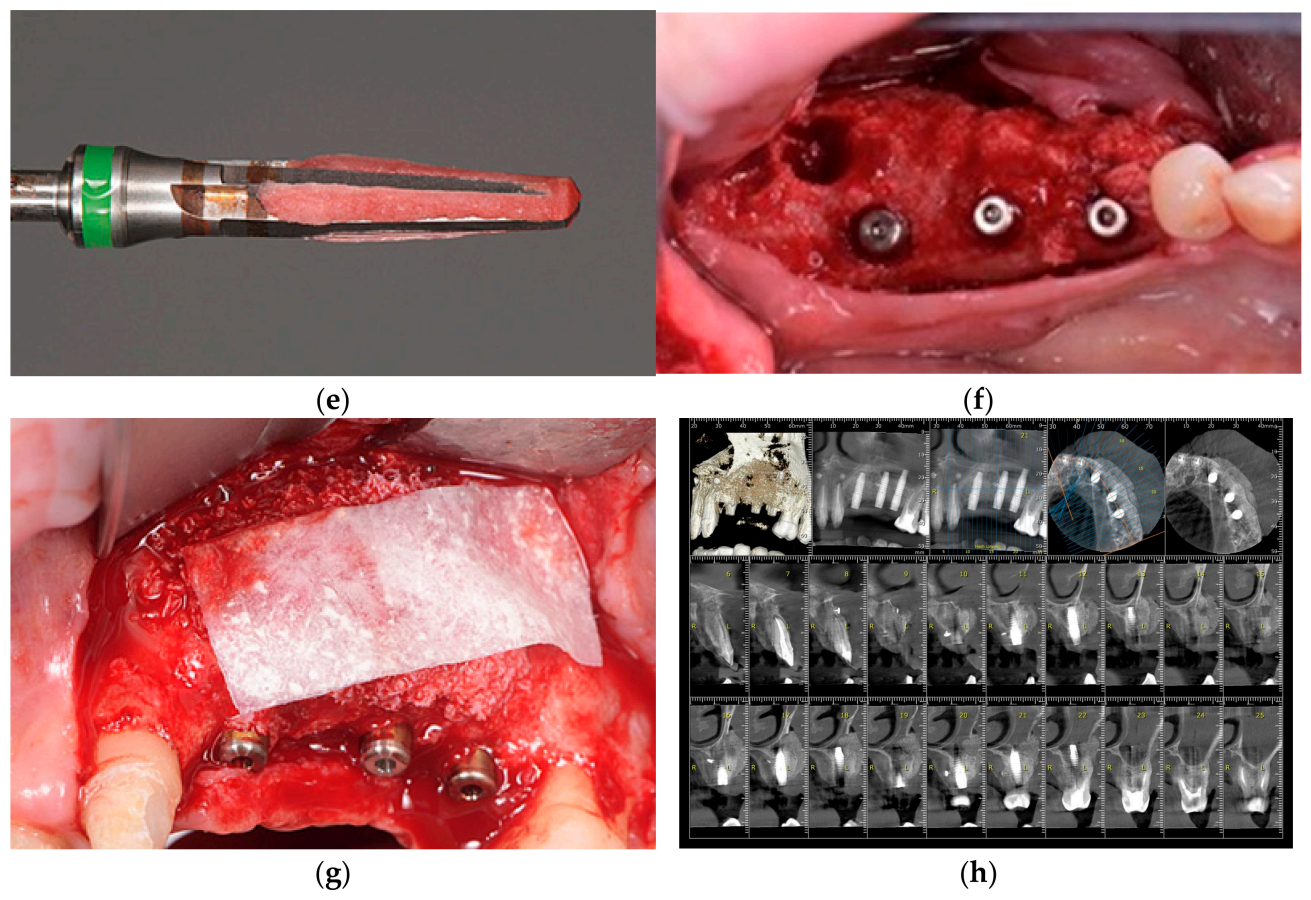
Representative clinical and radiographic stages of implant placement following LRA. (**a**) Intraoperative view at re-entry of the mandibular site after healing; (**b**) CBCT demonstrating increased ridge width; (**c**) Re-entry of regenerated maxillary sites after healing; (**d**) CBCT obtained five months after augmentation, illustrating increased RBW; (**e**) Bone core biopsy harvested using a bone collector drill; (**f**) Implant placement in regenerated bone, with the adjacent bone core biopsy site; (**g**) Localized grafting of biopsy site; (**h**) Postoperative CBCT confirming implant positioning and regenerated ridge dimensions.

Three to five months after the initial surgery, CBCT imaging was performed to evaluate the augmented alveolar ridge. Clinical and radiographic assessments confirmed sufficient bone regeneration to permit placement of standard-diameter implants, with ridge widths ranging approximately from 8 to 12 mm (Figure 4a–d). Representative cases from both the mandible and maxilla were included to illustrate the clinical applicability of the technique across different anatomical conditions (Figure 4a,c).

Preoperative preparation followed the same protocol as described for the first surgical stage. A trapezoidal mucoperiosteal incision was performed to expose the grafted area and adjacent teeth. Flap elevation revealed a well-integrated and mature cortical bone layer in the augmented area (Figure 4a,c).

Implant sites were prepared according to the manufacturer’s protocol for standard-length, standard-diameter implants (Figure 4d). No additional bone condensation techniques, such as osteotome-mediated condensation or osseodensification, were performed during implant osteotomy preparation. Implant sites within regenerated bone were prepared using the manufacturer’s conventional drilling protocol. This approach was intentionally selected to preserve the structural integrity and vascular architecture of the regenerated tissue, avoiding excessive mechanical compression that could potentially compromise bone biology or alter clinical interpretation. The implants placed in this study were Prama^®^ dental implants (Sweden & Martina, Due Carrare, Italy).

After implant placement, bone core biopsies were collected from the regenerated sites using a 5 mm autogenous bone collector bur (Sweden & Martina, Due Carrare, Italy) (Figure 4e). The region of interest (ROI) selection of bone biopsy comprised the entire central biopsy section representing the regenerated augmented area, while excluding sectioning artifacts and, where identifiable, pre-existing native cortical bone margins not representative of regenerated tissue. This approach was selected to maximize representative assessment of graft remodeling and tissue integration.

Following implant placement (Figure 4e), the biopsy sites were re-grafted with small-particle collagenated porcine-derived xenograft and covered with a resorbable collagen membrane (BioCover™, Purgo Biologics Inc., Gyeonggi-do, Republic of Korea) (Figure 4g). The surgical site was subsequently closed with sutures, and a postoperative CBCT scan was obtained to confirm implant positioning and graft adaptation (Figure 4h).

Postoperative care followed the same protocol as described for the previous surgery.

#### 2.3.4. Soft Tissue Surgery

**Figure 5:**
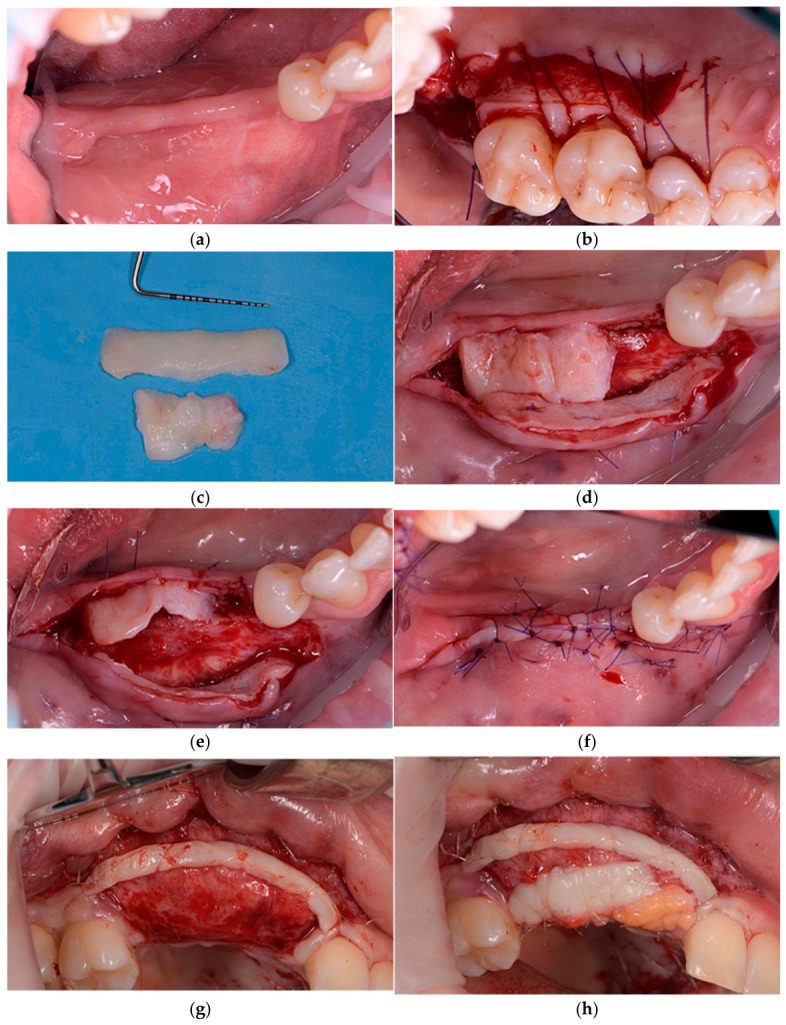
Representative soft tissue augmentation procedures following implant placement. (**a**) Preoperative peri-implant soft tissue conditions; (**b**,**c**) Palatal connective tissue graft harvesting; (**d**–**f**) Mandibular closed connective tissue graft stabilization and flap closure; (**g**,**h**) Maxillary vestibuloplasty and connective tissue graft placement for soft tissue volume enhancement.

Three months after implant placement, a soft tissue augmentation procedure was performed to improve peri-implant mucosal thickness and quality (Figure 5a). Subepithelial connective tissue grafts were harvested from the palate and trimmed to the desired dimensions (Figure 5b,c).

Two different approaches for graft stabilization were applied depending on the anatomical site. In the mandibular posterior region, a de-epithelialized connective tissue graft was used. A split-thickness (supraperiosteal) flap was prepared, along with a mucosal flap. One graft was stabilized onto the periosteum (Figure 5d), while a second graft was positioned and sutured to the inner aspect of the mucosal flap (Figure 5e). The flap was coronally advanced to achieve tension-free primary closure and closed healing (Figure 5f).

In the maxillary anterior and premolar regions, a split-thickness supraperiosteal flap was prepared and apically repositioned, resulting in a vestibuloplasty effect (Figure 5g). Two epithelialized connective tissue grafts were placed directly onto the periosteum: a strip graft and a free gingival graft to increase soft tissue volume and stability (Figure 5h). The surgical site was left partially uncovered, allowing healing by secondary intention.

These site-specific variations were adopted to account for differences in soft tissue thickness, vascularization, and anatomical features between the mandible and maxilla.

#### 2.3.5. Prosthetic Rehabilitation

Six months after implant placement, prosthetic rehabilitation was initiated. The implants were surgically uncovered, and healing abutments were placed for a period of two weeks to allow soft tissue maturation and peri-implant contouring.

**Figure 6:**
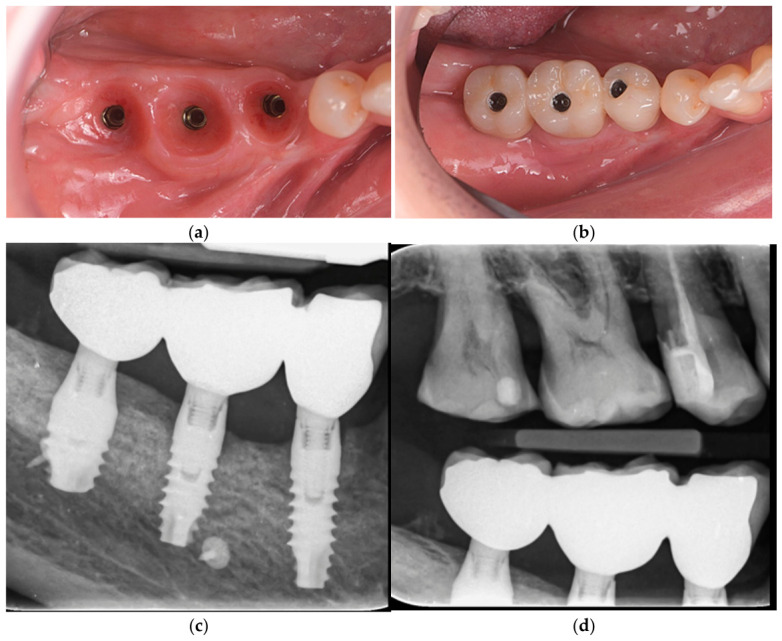
Representative prosthetic rehabilitation outcomes. (**a**) Peri-implant soft tissue maturation at prosthetic stage; (**b**) Occlusal view of the screw-retained implant-supported restorations; (**c**,**d**) Radiographic confirmation of implant positioning and marginal adaptation.

At the time of prosthetic intervention, peri-implant soft tissues exhibited favorable conditions, with adequate thickness and stability (Figure 6a). Subsequently, screw-retained porcelain-fused-to-metal crowns were delivered after an additional one week (Figure 6b).

Occlusion was carefully adjusted to ensure proper load distribution. Postoperative radiographs confirmed accurate fit and proper seating of the restorations on the implants (Figure 6c,d).

### 2.4. Biopsy Collection and Processing

Bone biopsies were not routinely included in the standard protocol for bone augmentation at this institution. In these cases, core bone biopsy collection was clinically indicated during implant osteotomy due to severe alveolar atrophy and the necessity to evaluate bone quality before standard implant placement. Histomorphometric analysis quantified new bone formation, residual graft material, and connective tissue (CT), defined as follows:

- New bone (NB): mineralized trabecular tissue containing viable osteocytes within lacunae, osteoblastic lining when present, and evidence of active matrix deposition.
- Residual graft material (BSM): acellular mineralized biomaterial particles distinguishable from native or newly formed bone by morphology, staining behavior, and structural characteristics.
- Connective tissue (CT): non-mineralized stromal tissue including fibrous matrix, vascular structures, and cellular soft tissue components.

All patients were thoroughly informed and provided written consent for the biopsy procedure and for the scientific use of anonymized tissue samples. Biopsies were performed solely for research purposes, and no additional surgical intervention or associated morbidity was incurred.

Biopsy specimens were obtained during implant placement from the buccal wall adjacent to the implant site preparation. They consisted of newly formed bone together with residual xenograft particles, while native cortical bone was excluded.

Following retrieval, specimens were immediately fixed in 10% buffered formalin for 48 h. Decalcification was carried out using 10% tris-buffered ethylenediaminetetraacetic acid (EDTA; Carl Roth, Karlsruhe, Germany) at 37 °C for seven days with the aid of an ultrasonic decalcification system (Medite, Dietikon, Switzerland). Once decalcified, samples underwent dehydration through ascending concentrations of ethanol and were subsequently cleared in xylene.

Each specimen was embedded longitudinally in paraffin, and serial sections of 2–4 μm thickness were obtained from the central portion using a microtome (Leica, Wetzlar, Germany). Standard histological processing included staining with hematoxylin and eosin (H&E), Masson–Goldner trichrome, and tartrate-resistant acid phosphatase (TRAP) to evaluate tissue morphology, mineralized structures, and osteoclastic activity.

TRAP staining was interpreted descriptively within the limitations of the available archival histological material. As this retrospective study relied on previously processed laboratory specimens, additional negative control sections were not available.

### 2.5. Histological Evaluation

Histomorphometric assessment was performed according to a standardized protocol using a research-grade digital microscopy system in combination with NIS-Elements software (version 5.21; Nikon, Tokyo, Japan) to ensure methodological consistency and analytical precision. Digital images covering the entire implantation region, including bone substitute material and peri-implant tissues, were obtained with a DS-Fi1 digital camera (Nikon, Tokyo, Japan) mounted on an Eclipse 80i microscope (Nikon, Tokyo, Japan) equipped with an automated scanning stage (Prior, Rockland, MA, USA), selected for its reliability in high-resolution imaging and quantitative analysis.

Image datasets were acquired at ×200 magnification to capture essential morphological details. Representative higher-magnification images of relevant histological features were extracted using Photoshop (Adobe, San Jose, CA, USA) to illustrate key findings.

An experienced oral pathologist, independent of the surgical procedures, evaluated the histological sections to reduce potential bias. Although a formal blinded assessment was not implemented due to the retrospective exploratory design and descriptive nature of this pilot case series, the evaluator was not informed of the specific clinical outcomes during microscopic analysis. This approach aimed to partially reduce interpretive bias; however, the absence of formal blinding is acknowledged as a methodological limitation.

Because this retrospective pilot case series relied on previously processed archival histological specimens and primarily descriptive analysis, formal inter-observer and intra-observer reliability assessments were not performed.

Histomorphometric measurements included the percentages of newly formed bone, residual graft material, and connective tissue within the region of interest, as these parameters serve as key quantitative indicators of tissue response and material integration. Histomorphometric analysis was performed as a two-dimensional, area-based descriptive evaluation of histological sections using digital microscopy software. Measurements were not extrapolated into volumetric three-dimensional calculations.

### 2.6. Postoperative and Follow-Up Protocol

Patients were followed according to a scheduled postoperative protocol at 24 h, one week, one month, three months, and six months after surgery. An immediate postoperative CBCT scan was performed to verify graft position.

RBW and postoperative ridge width measurements were obtained from standardized cross-sectional CBCT slices oriented perpendicular to the long axis of the alveolar ridge at the planned implant recipient sites. Measurements were performed at anatomically consistent reference points corresponding to the center of the edentulous ridge and intended implant position, allowing comparison between preoperative and postoperative ridge dimensions.

Because this study was retrospective and based on routine clinical imaging records rather than a prospectively standardized radiographic protocol, formal blinded examiner analysis and intra-examiner reproducibility assessments were not performed. This limitation has now been explicitly acknowledged in the revised manuscript.

At the first postoperative week, patients underwent clinical evaluation focusing on pain levels, soft tissue healing, swelling, infection, and suture condition. Only mild discomfort was reported, which was successfully managed with the prescribed analgesics. No cases of infection, flap dehiscence, or abnormal swelling were observed.

Follow-up examinations at one and three months confirmed stable healing, favorable soft tissue maturation, and the absence of postoperative complications.

After a healing interval of 3–5 months, standard-diameter implants were placed in the augmented sites.

Six months after implant placement and soft tissue augmentation, the treated sites demonstrated sufficient bone volume and adequate width of keratinized mucosa. Screw-retained porcelain-fused-to-metal implant-supported crowns were subsequently delivered. All implants exhibited excellent primary and secondary stability, with no mobility, patient discomfort, or clinical signs of inflammation. Both clinical and radiographic evaluations confirmed stable marginal bone levels and the absence of peri-implant pathology.

**Figure 7:**
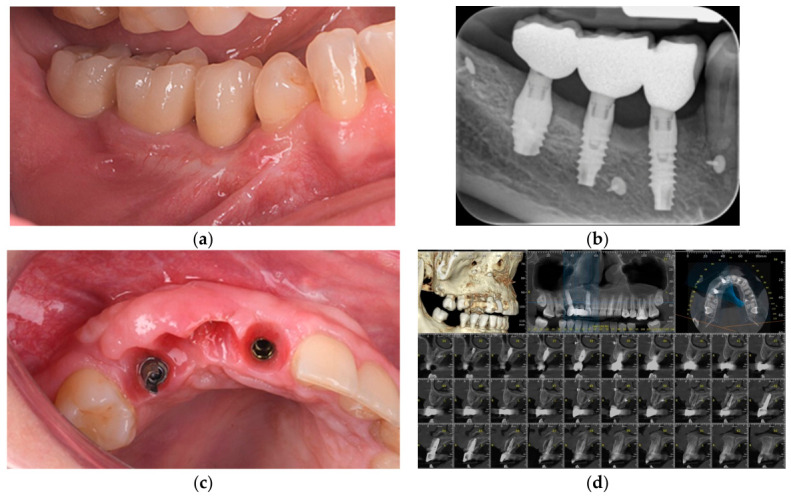
Representative long-term clinical and radiographic follow-up findings. (**a**) Clinical follow-up view at 55 months, showing stable peri-implant soft tissue conditions; (**b**) Periapical radiograph at 55 months, showing stable marginal bone levels; (**c**) Clinical follow-up view at 46 months, illustrating stable peri-implant soft tissue architecture after removal of the prosthetic restoration; (**d**) CBCT scan at 46 months, confirming maintained ridge dimensions and augmented bone stability.

Thereafter, annual follow-up visits were conducted to monitor implant success, peri-implant bone stability, and patient satisfaction. Over a mean long-term follow-up period of 54.2 months, no implant failures were observed. Marginal bone levels remained stable within acceptable limits, peri-implant soft tissues remained healthy, and the augmented ridge width demonstrated sustained dimensional stability, supporting favorable long-term outcomes within the limitations of this pilot cohort (Figure 7a–d).

This protocol enabled consistent assessment of clinical healing, graft integration, and implant survival across all three cases. The case series was conducted and reported in accordance with the CARE (CAse REport) guidelines, as summarized in Table 1.

## 3. Results

### 3.1. Clinical Outcomes

Three patients (one male and two females; mean age, 43.33 years; range, 34–62 years) underwent LRA using particulate collagenated porcine-derived xenograft (0.25–1.00 mm) with particulate autogenous bone and staged implant placement (Table 2).

No intraoperative or postoperative complications were observed, and all surgical sites demonstrated uneventful healing.

Radiographic evaluation revealed substantial horizontal bone gain, with mean ridge width increasing from 2.33 mm preoperatively to 11.67 mm postoperatively. All implants achieved satisfactory primary stability, and screw-retained prosthetic crowns were delivered six months after implant placement.

During long-term follow-up (mean: 54.2 months; range: 50.7–58.5 months), all implants remained functional, with no evidence of implant failure, mobility, or peri-implant infection.

### 3.2. Histological and Histomorphometric Outcomes

Bone biopsies were harvested at 4, 5, and 3 months post-augmentation (Table 3). Biopsies were intentionally harvested from areas considered to present reduced regenerative potential, such as the posterior mandibular and anterior maxillary regions, in order to assess bone formation under more challenging biological conditions.

All specimens demonstrated newly formed trabecular bone (NB), integration of the xenogenic bone substitute material (BSM), and clear evidence of vascularization.

Biopsy C1221-4 (4 months post-augmentation)

Histological analysis demonstrated a well-preserved longitudinal specimen, with residual native bone identifiable in the apical and lateral regions. Newly formed trabecular bone (NB) and residual bone substitute material (BSM) were distributed throughout the biopsy, with a greater concentration of BSM in the crestal region. Autogenous bone material (ABM) and NB were observed both longitudinally and axially, while NB was distinguishable by its higher cellularity and lower mineral density.

**Figure 8:**
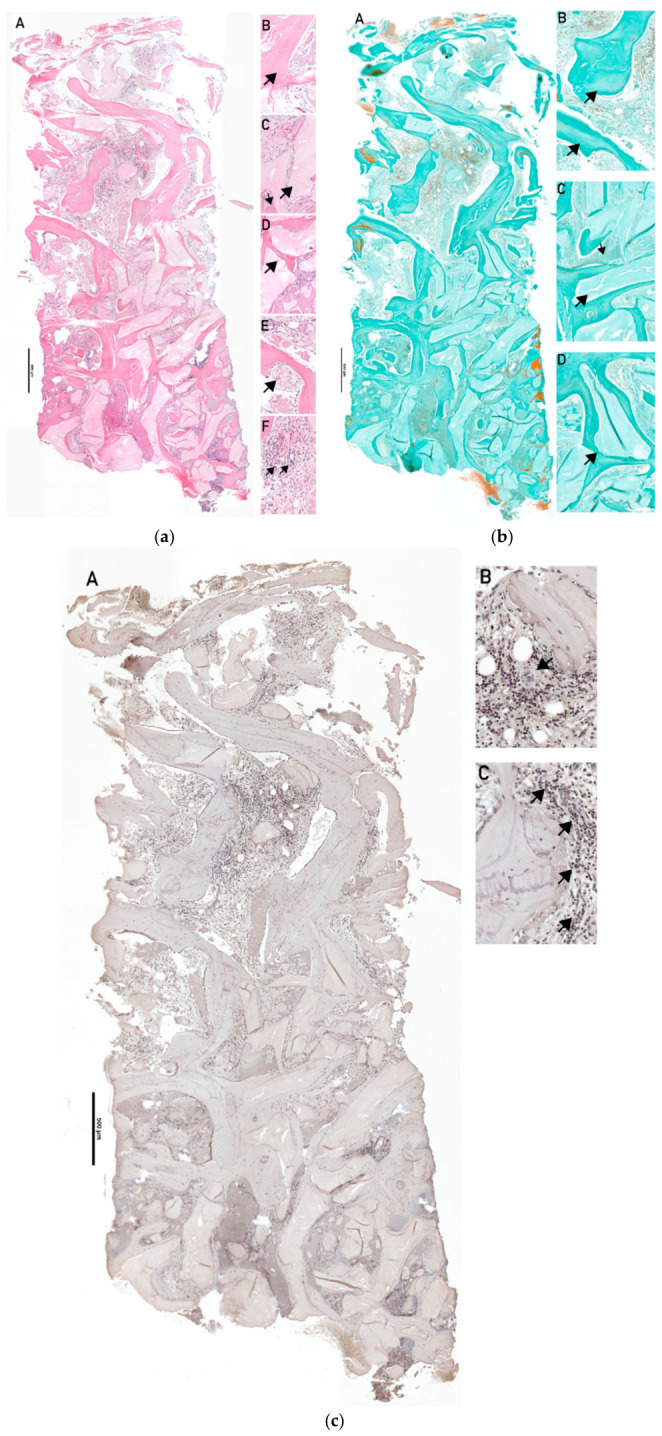
(**a**) Histological evaluation of biopsy C1221-4 (H&E staining). (A) Total biopsy overview (scale bar: 500 µm). (B) Newly formed bone (NB). (C) Residual bone substitute material (BSM; large arrow) and autogenous bone material (ABM; small arrow). (D) Hybrid bone formation. (E) Vascularization. (F) Lymphocyte infiltration within connective tissue (CT). (**b**) Histological evaluation of biopsy C1221-4 (Masson–Goldner staining). (A) Total biopsy overview (scale bar: 500 µm). (B) Mineralized newly formed bone (NB) surrounded by connective tissue (CT). (C) Residual bone substitute material (BSM; large arrow) and autogenous bone material (ABM; small arrow). (D) Hybrid bone formation. (**c**) TRAP immunohistological staining evaluation of biopsy C1221-4. Small arrows: MNCs TRAP-positive. (A) Total biopsy overview (scale bar: 500 µm). (B) Representative TRAP-negative multinucleated giant cell. (C) Representative TRAP-positive mononuclear cell infiltration. Staining: Anti-TRAP (tartrate-resistant acid phosphatase) immunohistological staining of paraffin histological sections.

Vascularization was evident within the connective tissue, particularly in the apical region, where small blood vessels with early endothelial wall formation were observed, indicating ongoing maturation (Figure 8a,b).

Mild lymphocyte infiltration was detected locally, along with TRAP-negative multinucleated giant cells (MNGCs) at the interface between BSM and connective tissue (Figure 8c). These findings suggest active remodeling and early-stage biomaterial degradation without adverse inflammatory response.

Overall, the biopsy demonstrated favorable integration of the xenograft material, active bone formation, and physiological remodeling processes.

Biopsy C-0622-1 (5 months post-augmentation)

A complete, although relatively small, longitudinal biopsy specimen was available for analysis. No residual native bone was identified within the sample. The xenogenic bone substitute material (BSM) appeared as isolated mineralized structures distributed throughout the biopsy, while autogenous bone could not be consistently distinguished.

Newly formed trabecular bone (NB), characterized by viable osteocytes within the matrix, was observed in both longitudinal and axial orientations. Histomorphometric estimation indicated approximately 20% newly formed bone, 20% residual graft/autogenous bone, and 60% connective tissue.

**Figure 9:**
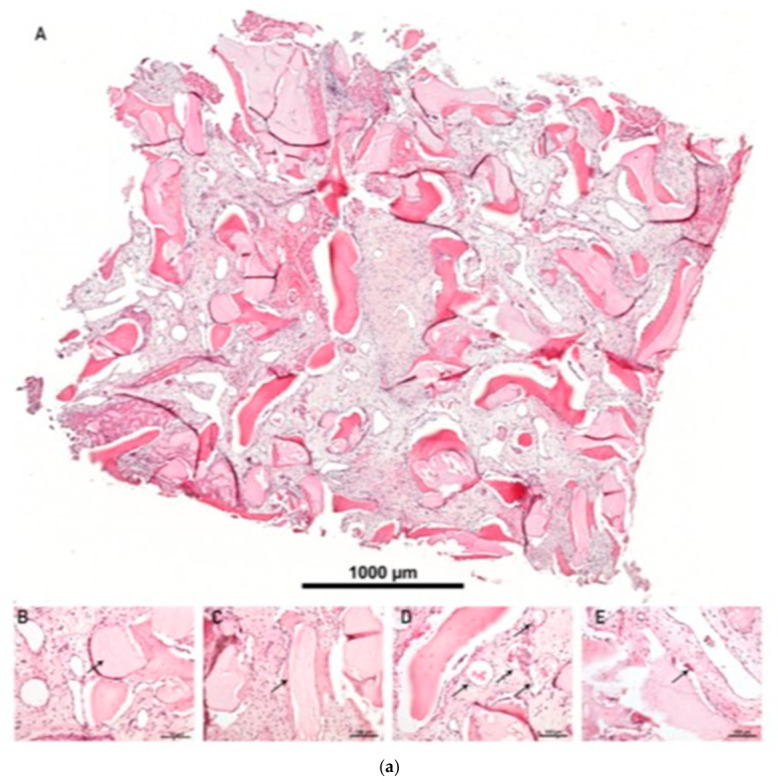

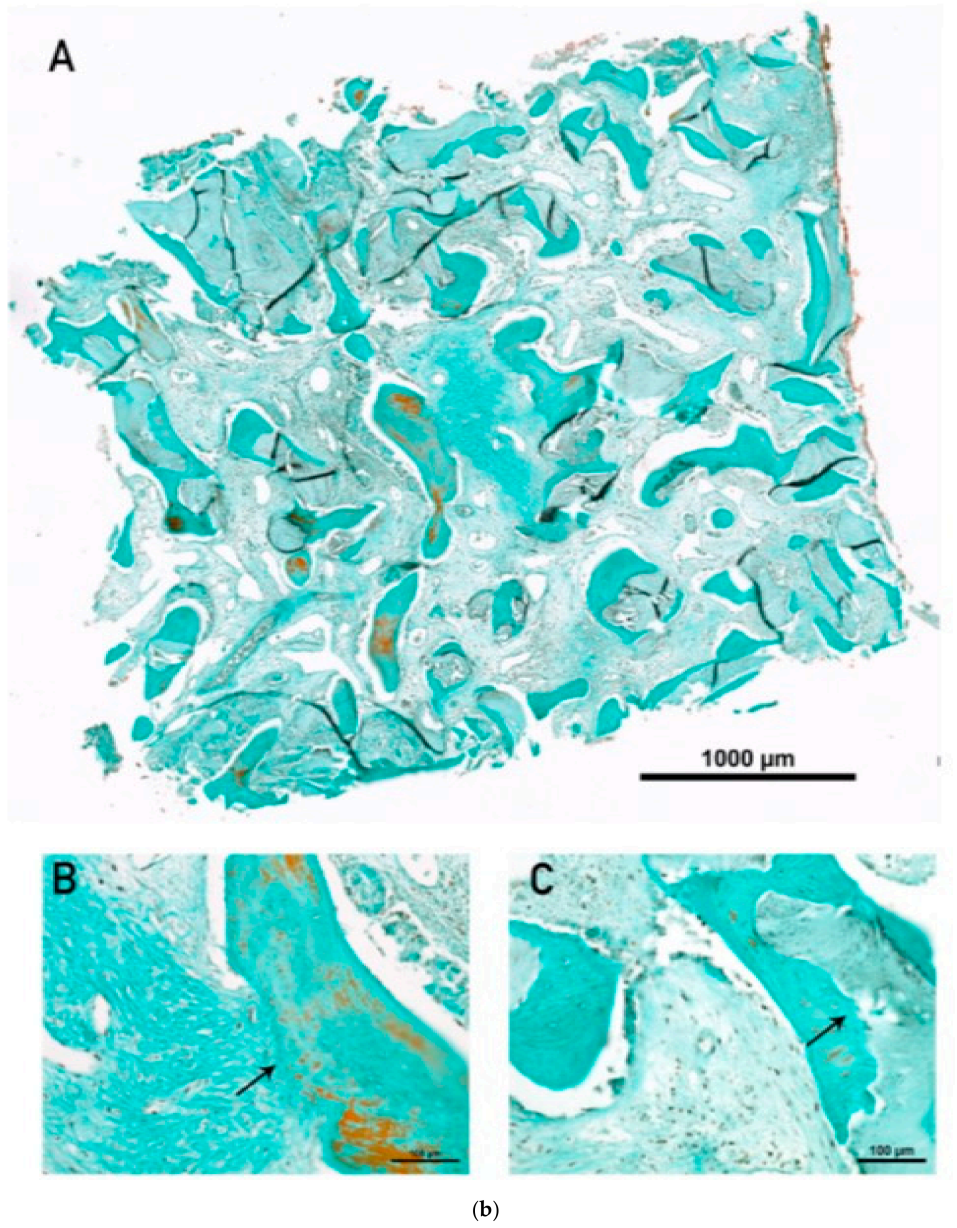

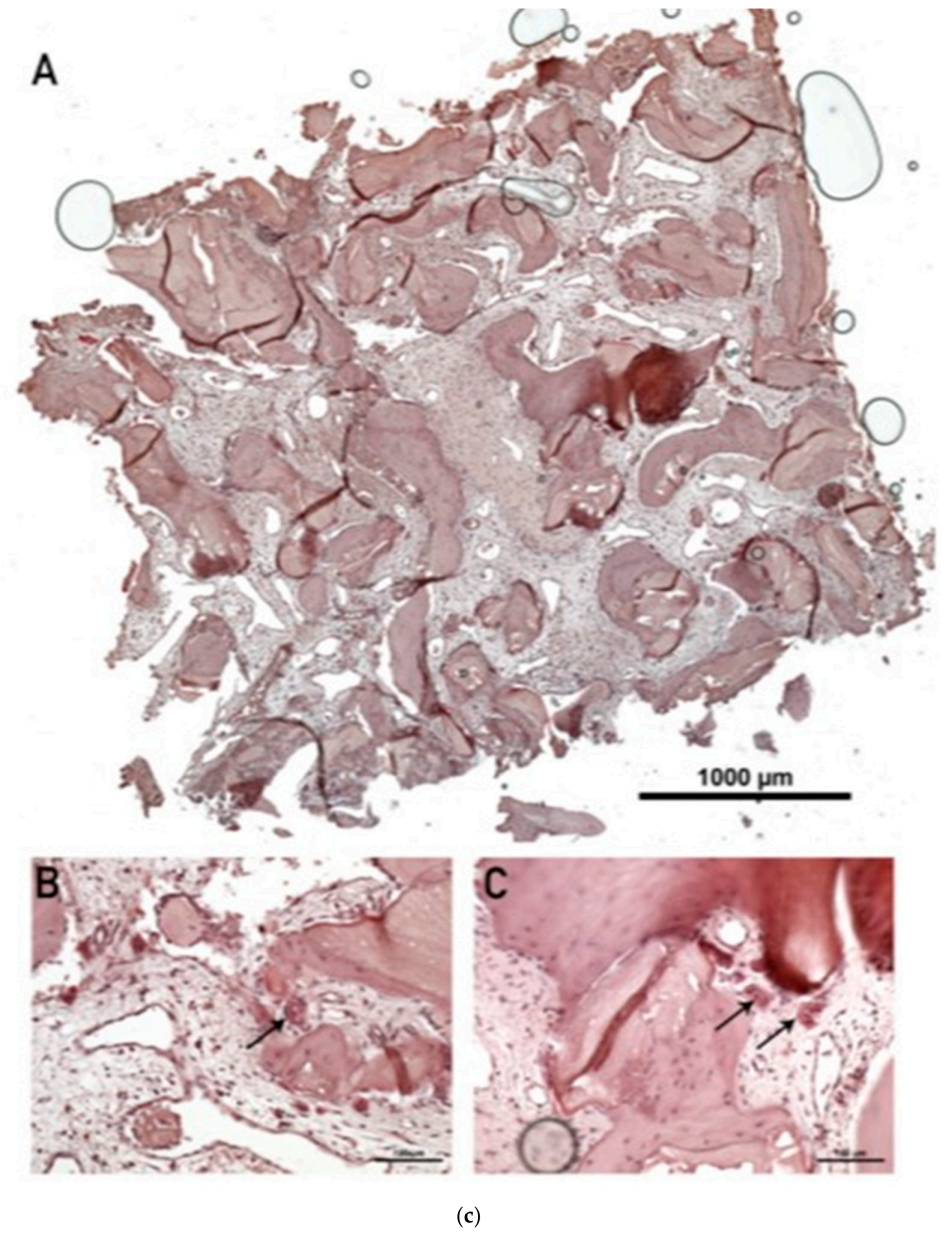
(**a**) Histological evaluation of biopsy C0622-1 (H&E staining). (A) Total biopsy overview (scale bar: 1000 µm). (B) Residual bone substitute material (BSM) and newly formed bone (NB). (C) Hybrid bone formation. (D) Vascularization. (E) Multinucleated giant cell (MNGC). Small arrows indicate relevant areas and/or structures. B: residual BSM and NB formation, C: hybrid bone formation, D: vessel formation, and E: MNGC. (**b**) Histological evaluation of biopsy C0622-1 (Masson–Goldner staining). (A) Total biopsy overview (scale bar: 1000 µm). (B) Mineralized bone structures. (C) Newly formed bone (NB) at the surface of residual bone substitute material (BSM). Small arrows indicate relevant areas and/or structures. (B): mineralized bony structures, (C): NB formation at BSM. (**c**) TRAP immunohistological staining evaluation of biopsy C0622-1. (A) Total biopsy overview (scale bar: 1000 µm). (B,C) Representative residual bone substitute material (BSM) surfaces demonstrating TRAP-positive multinucleated giant cell (MNGC) accumulation. Staining: Anti-TRAP (tartrate-resistant acid phosphatase) immunohistological staining of paraffin histological sections. Small arrows indicate relevant areas and/or structures. (B,C): representative images for BSM surface with TRAP-positive MNGC cell accumulation.

Vascularization was well developed, with blood vessels evenly distributed throughout the connective tissue. The vessels appeared relatively large, with mature endothelial wall formation, indicating advanced tissue maturation (Figure 9a).

New bone formation was clearly evident along the surface of BSM particles, with distinct features of hybrid bone formation resulting from the incorporation of xenograft granules into the newly generated bone matrix (Figure 9b). The BSM was well integrated within both newly formed bone and connective tissue compartments, whereas the autogenous bone appeared largely resorbed.

No significant inflammatory response or pathological lymphocyte infiltration was observed. However, TRAP-positive MNGCs were present at the interface between BSM and connective tissue (Figure 9c), indicating a mild foreign-body reaction associated with ongoing biomaterial degradation and remodeling.

Overall, the biopsy demonstrated active bone regeneration, good integration of the xenograft material, and advanced vascular maturation, with ongoing but controlled biomaterial resorption.

Biopsy C-0622-2 (3 months post-augmentation)

The biopsy consisted of two fragments (C-0622-2.1 and C-0622-2.2), which were analyzed separately due to differences in tissue composition and maturation.

In fragment C-0622-2.1, no residual native bone was identified. The xenogenic bone substitute material (BSM) was evenly distributed throughout the sample as acellular mineralized structures. Newly formed trabecular bone (NB), characterized by viable osteocytes, was also evenly distributed. Histomorphometric estimation revealed approximately 10% newly formed bone, 30% residual graft material, and 60% connective tissue.

In fragment C-0622-2.2, a more heterogeneous structure was observed. Both residual BSM and autogenous bone were present, with BSM predominantly located in the upper regions and autogenous bone in the lower regions. New bone formation was detected throughout the fragment. Histomorphometric estimation indicated approximately 15% newly formed bone, 10% residual graft material, 15% autogenous bone, and 40% connective tissue.

**Figure 10:**
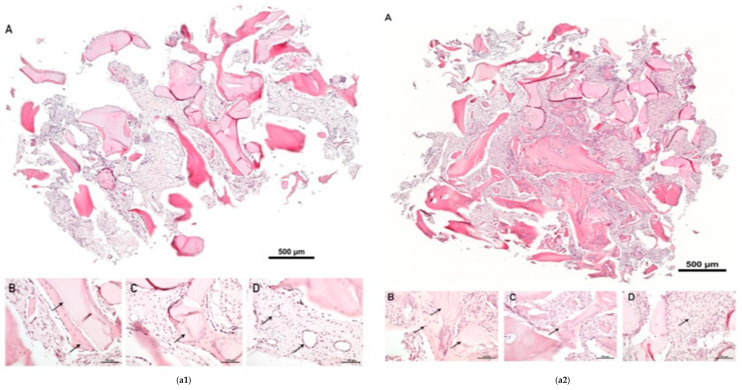

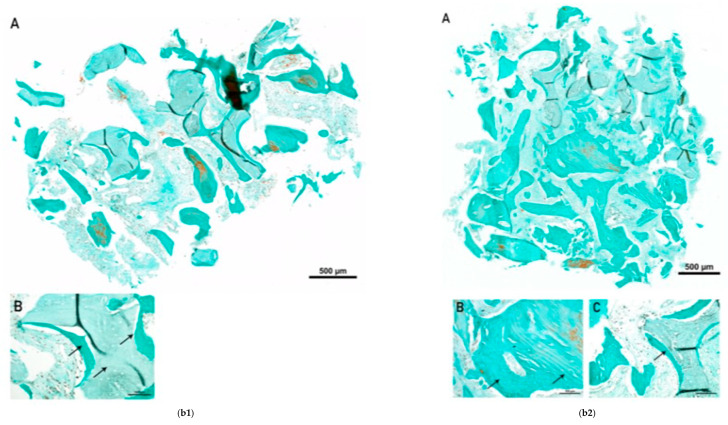

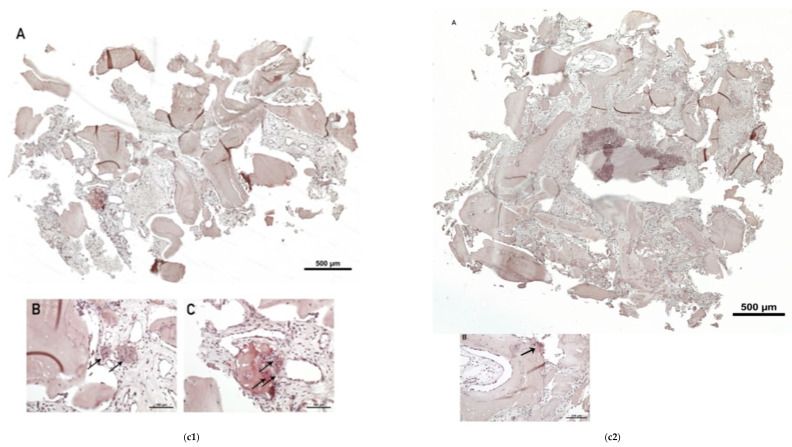
(**a1**) Histological evaluation of biopsy C0622-2.1 (H&E staining, fragment 1). (A) Total biopsy overview. (B) Newly formed bone (NB) and hybrid bone formation. (C) Residual bone substitute material (BSM). (D) Vascularization. Small arrows indicate relevant areas and/or structures. (B): NB and hybrid bone formation, (C): residual BSM, and (D): vessel formation. (**a2**) Histological evaluation of biopsy C0622-2.2 (H&E staining, fragment 2). (A) Total biopsy overview. (B) Residual autogenous bone material (ABM) and newly formed bone (NB). (C) Hybrid bone formation. (D) Vascularization. Small arrows indicate relevant areas and/or structures. (B): residual autogenous bone and new bone formation, (C): hybrid bone formation, and (D): vessel formation. (**b1**) Histological evaluation of biopsy C0622-2.1 (Masson–Goldner staining, fragment 1). (A) Total biopsy overview (scale bar: 500 µm). (B) Hybrid bone formation with newly formed bone (NB) on the surface of residual bone substitute material (BSM). Small arrows indicate hybrid bone formation. (**b2**) Histological evaluation of biopsy C0622-2.2 (Masson–Goldner staining, fragment 2). (A) Total biopsy overview (scale bar: 500 µm). (B) Newly formed bone (NB) on residual mineralized autogenous bone structures. (C) Hybrid bone formation within residual bone substitute material (BSM). Small arrows indicate (B): NB and (C): hybrid bone. (**c1**) TRAP immunohistological staining evaluation of biopsy C0622-2.1 (fragment 1). (A) Total biopsy overview (scale bar: 500 µm). (B,C) Representative multinucleated giant cells (MNGCs). (C) TRAP-positive multinucleated cells associated with degradation of residual bone substitute material (BSM). Staining: Anti-TRAP (tartrate-resistant acid phosphatase) immunohistological staining of paraffin histological sections. Small arrows indicate relevant areas and/or structures. (B,C): examples of MNGCs; (C): TRAP-positive foreign body cells degrading residual BSM. (**c2**) TRAP immunohistological staining evaluation of biopsy C0622-2.2 (fragment 2). (A) Total biopsy overview (scale bar: 500 µm). (B) Representative TRAP-positive multinucleated giant cell (MNGC) on the surface of residual autogenous bone material (ABM). Staining: Anti-TRAP (tartrate-resistant acid phosphatase) immunohistological staining of paraffin histological sections. Small arrows indicate (B): example for TRAP-positive MNGC on surface of residual autogenous bone particles.

New bone formation was observed on the surface of both BSM and autogenous bone, with evidence of hybrid bone formation, confirming the osteoconductive properties of the xenograft and its integration into the newly formed bone matrix (Figure 10(a1,b1); Figure 10(a2,b2)).

Vascularization was evident in both fragments, although with different maturation patterns. In fragment C-0622-2.1, blood vessels were more evenly distributed and appeared larger, with well-defined endothelial walls, indicating advanced vascular maturation (Figure 10(a1)). In contrast, fragment C-0622-2.2 showed predominantly smaller vessels with early wall formation, consistent with a less mature stage of angiogenesis (Figure 10(a2)).

TRAP-positive and TRAP-negative MNGCs were observed at the interface between the BSM and connective tissue in fragment C-0622-2.1, suggesting ongoing remodeling activity; however, these findings should be interpreted cautiously given the descriptive nature of the analysis (Figure 10(c1)). In fragment C-0622-2.2, only a limited number of TRAP-positive cells were detected (Figure 10(c2)), suggesting a less advanced resorptive activity in this region.

Both fragments demonstrated active bone regeneration and good integration of the xenograft material, with ongoing remodeling processes. The observed heterogeneity reflects different stages of healing within the augmented site, with simultaneous bone formation and biomaterial degradation.

Histological findings varied according to the healing time rather than patient age, with early-stage samples showing higher connective tissue content and later samples demonstrating increased bone organization and vascular maturation. Differences in histological outcomes were also influenced by the anatomical location, with the mandibular posterior site showing a higher proportion of newly formed bone compared to maxillary anterior sites at similar healing intervals.

All biopsies of three patients demonstrated progressive bone formation, vascularization, and integration of the xenograft material, with increasing evidence of remodeling over time.

## 4. Discussion

The present pilot case series assessed the clinical, radiographic, and histological outcomes of LRA performed with a collagenated porcine-derived xenograft in combination with autogenous bone. Within the limitations of this study, the findings descriptively suggest substantial horizontal bone gain, successful implant placement, and favorable long-term stability, supported by both histological and histomorphometric evidence.

From a clinical perspective, all augmentation sites healed uneventfully, with no intraoperative or postoperative complications, and all implants remained functional during a mean follow-up period of 54.2 months. Although extended postoperative antibiotic prophylaxis was routinely used in this cohort, evolving antibiotic stewardship recommendations increasingly support minimizing antibiotic exposure where clinically appropriate. Therefore, the protocol described here should be interpreted within its retrospective clinical context rather than as evidence of an optimized antimicrobial regimen.

These results are consistent with the existing literature demonstrating high survival rates of implants placed in regenerated bone following guided bone regeneration (GBR) procedures [5,7,9,33]. Systematic reviews suggest that GBR represents a well-documented and clinically established approach for horizontal ridge augmentation, with implant survival rates comparable to those placed in native bone [34,35,36,37]. However, outcomes remain influenced by defect morphology, surgical technique, and biomaterial characteristics.

Radiographically, a marked increase in ridge width was observed (mean gain ≈ 9.67 mm), allowing the placement of standard-diameter implants in all cases. Although this gain appears substantial, it should be interpreted carefully due to the limited sample size and the absence of standardized volumetric analysis. Nevertheless, the magnitude of horizontal augmentation reported here is in line with previous clinical studies and systematic reviews reporting mean horizontal gains of 3–6 mm using particulate GBR techniques [7,30,34,35,38,39,40,41]. Due to the descriptive design, no definitive conclusions can be drawn regarding the relative contribution of graft composition or surgical technique. Although bone condensation techniques may improve primary stability in selected native low-density bone scenarios, their routine application in regenerated bone remains controversial [42,43]. Excessive condensation within recently regenerated grafted sites may disrupt newly formed trabecular architecture, impair vascularization, or introduce unnecessary mechanical trauma [42,43]. In the present study, conventional osteotomy preparation was intentionally used to preserve the biological integrity of the regenerated bone and to allow more direct evaluation of graft-derived regenerative outcomes.

The histological and histomorphometric findings provided important data on the biological behavior of the grafting material. All biopsies demonstrated newly formed trabecular bone, residual graft material, and well-vascularized connective tissue, reflecting different stages of bone regeneration. Importantly, new bone formation was consistently observed on the surface of both xenogenic and autogenous particles, with clear evidence of hybrid bone formation. These descriptive observations are consistent with the osteoconductive role previously reported for collagenated porcine-derived xenografts [23,24,25].

A relevant aspect of the present study is the use of TRAP staining to evaluate osteoclastic activity at the graft–bone interface. The presence of TRAP-positive MNGCs suggests active biomaterial resorption and ongoing remodeling [44,45,46]. Although TRAP-positive multinucleated giant cells may indicate active biomaterial-associated remodeling, their biological significance remains complex. TRAP activity may reflect physiological scaffold degradation and osteoclastic remodeling, but may also represent aspects of foreign body response depending on cellular phenotype, persistence, and inflammatory context. Therefore, the presence of TRAP-positive cells should not be interpreted exclusively as favorable remodeling, but rather as part of a broader host biomaterial interaction requiring cautious interpretation. The long-term persistence of residual graft particles and associated multinucleated giant cells warrants further investigation regarding their implications for biomaterial turnover and tissue stability. This supports the concept that the material is not biologically inert, but rather participates in a dynamic process characterized by simultaneous bone formation and graft degradation [47].

These findings are consistent with previous observations reported in sinus floor augmentation using the same xenograft, but without autogenous bone, where TRAP-positive activity indicated ongoing remodeling processes [48].

In contrast to bovine-derived xenografts, which are known for their slow resorption and long-term persistence, porcine-derived xenografts may exhibit a more balanced remodeling profile [24,37,49,50,51]. Systematic reviews have highlighted that the ideal grafting material should combine structural stability with controlled resorption, allowing gradual replacement by vital bone [36,52,53,54]. The present histological observations descriptively indicate ongoing bone formation and biomaterial-associated remodeling; however, comparative conclusions regarding biological behavior relative to other grafting materials are beyond the scope of this pilot case series.

The temporal evolution observed in the biopsies further supports this interpretation. Early-stage samples (3 months) showed higher connective tissue content and lower bone fraction, whereas later samples (4–5 months) demonstrated increased bone formation, improved trabecular organization, and more mature vascularization. This progression is consistent with the physiological sequence of bone healing described in both preclinical and clinical studies [44,55]. Differences observed between specimens collected at varying healing intervals may reflect distinct stages of tissue maturation. However, these descriptive findings require validation in larger standardized cohorts.

Another important observation is that biopsies were intentionally obtained from anatomical regions with reduced regenerative potential, such as the posterior mandible and anterior maxilla [56]. Despite these challenging conditions, favorable bone formation and graft integration were achieved. This finding suggests that the applied GBR protocol may be effective even in less predictable clinical scenarios, although this hypothesis requires confirmation in larger controlled studies. Another relevant observation is that substantial bone regeneration was achieved regardless of patient age, including in the oldest patient (62 years). This finding may indicate that the regenerative potential of the applied biomaterial is maintained across a broader patient population, although further studies are required to confirm this observation.

When compared with alternative techniques for horizontal ridge augmentation, such as ridge splitting or autogenous block grafting, the present approach offers several advantages. Ridge splitting is limited by the requirement for a minimum residual ridge width and carries a risk of buccal plate fracture, particularly in dense mandibular bone [57,58]. Autogenous block grafting, although considered the gold standard due to its osteogenic potential, is associated with increased surgical morbidity, donor-site complications, and unpredictable and variable resorption patterns, despite generally favorable clinical outcomes reported in the literature [37,59,60,61,62]. In contrast, GBR using particulate grafts allows better adaptation to defect morphology, reduced morbidity, and more favorable soft tissue management [7,16,34,35,36,63].

Alternative treatment strategies for horizontal ridge deficiency—including ridge preservation, immediate implant placement, narrow-diameter implants, blade implants, wedge-shape implants, ridge expansion, or graft-less prosthetic compensation—may reduce surgical morbidity in selected cases, such as extraction sockets or peri-implant gaps [64,65,66]. Blade implants may represent a potential treatment option in narrow ridges, particularly when combined with personalized design approaches [63]. However, they generally require approximately 1 mm of surrounding bone on each side, in addition to the implant width, resulting in a minimum RBW of at least 3 mm [67]. This anatomical requirement may still restrict their applicability in cases of severe horizontal atrophy [68]. Overall, the clinical use of these less invasive approaches is often restricted in the presence of severe horizontal ridge deficiency (residual bone width approximately 2–4 mm), where prosthetically driven implant positioning and long-term biomechanical stability may be compromised without substantial augmentation. In such advanced defects, LRA with staged implant placement remains one of the most predictable approaches for reconstructing adequate ridge dimensions to support conventional implant rehabilitation compared with less invasive alternatives [14,38,68]. Therefore, while minimally invasive strategies should be carefully considered during treatment planning, their indications must be balanced against anatomical limitations, prosthetic requirements, and long-term functional stability.

Although the combined use of autogenous bone and xenograft was associated with favorable descriptive outcomes in these cases, the present study design does not permit conclusions regarding synergistic regenerative effects. However, the combination of autogenous bone with a slowly resorbing xenograft might provide an interaction, balancing early biological activity with long-term structural stability. This outcome has been supported by previous studies and systematic reviews, which suggest that composite grafts may provide improved bone formation compared to single-material approaches [37,52,53,69].

From a clinical perspective, the present findings suggest that the use of a collagenated porcine-derived xenograft in combination with autogenous bone may represent a reliable strategy for lateral ridge augmentation, particularly in cases with limited ridge width and challenging vascular conditions. The observed balance between graft stability and controlled remodeling may contribute to maintaining augmented volume while allowing progressive replacement by vital bone, which is essential for long-term implant success. Furthermore, the presence of active TRAP-mediated remodeling indicates that the grafted site remains biologically dynamic, potentially enhancing adaptation to functional loading over time.

In addition to hard tissue regeneration, soft tissue management played a relevant role in the overall treatment outcome. The use of connective tissue grafting contributed to increased peri-implant mucosal thickness and improved soft tissue stability, factors that are known to be critical for long-term implant success and maintenance of marginal bone levels [70,71,72].

These aspects are particularly relevant in the context of peri-implant disease prevention, as previous clinical observations have emphasized the importance of soft tissue quality and stability in reducing the risk of peri-implant inflammation and disease progression [73].

## 5. Limitations

Despite these encouraging findings, the present study has several limitations. The most significant limitation is the small sample size (n = 3), which restricts generalizability and precludes statistical analysis. The highly selective inclusion criteria, particularly the requirement for biopsy availability and complete histological documentation, may also have introduced selection bias. In addition, the retrospective design and absence of a control group do not permit direct comparison with alternative grafting materials or augmentation techniques.

Although histological assessment was performed independently by an experienced oral pathologist, the absence of a formal blinded evaluation may have introduced interpretive bias. Furthermore, formal inter- and intra-observer reproducibility analyses were not available due to the retrospective, descriptive design, which may have reduced methodological robustness. Variability in anatomical sites and healing times at the time of biopsy collection may also have influenced histological outcomes. Histomorphometric analysis was performed on limited biopsy samples, which may not fully represent the entire augmented volume. Additionally, the lack of supplementary negative controls for TRAP staining may limit the definitive interpretation of staining specificity.

While some variability was observed between anatomical sites and healing stages, these findings should be interpreted cautiously. Given the exploratory design and limited cohort, observations regarding anatomical variability remain descriptive and do not allow definitive conclusions concerning site-specific regenerative behavior.

Radiographic assessment was based on retrospective linear CBCT measurements obtained from routine clinical imaging records rather than a prospectively standardized radiographic protocol. Although measurements were performed using anatomically consistent cross-sectional reference points, formal blinded examiner evaluation, intra-examiner reproducibility analysis, and standardized volumetric assessment were not available. Consequently, dimensional outcomes should be interpreted cautiously. Future prospective studies should incorporate calibrated radiographic protocols with three-dimensional volumetric analysis to improve measurement precision and reproducibility.

Additionally, the perioperative antibiotic regimen reflected institutional clinical practice during the treatment period and may not fully align with current evidence-based antimicrobial stewardship recommendations.

Future investigations should include larger patient cohorts, standardized volumetric analyses, and controlled comparisons between different biomaterials and treatment approaches. In particular, prospective randomized controlled trials integrating clinical, radiographic, and histological outcomes are necessary to better define the role of collagenated porcine-derived xenografts in lateral ridge augmentation.

## 6. Conclusions

Within the limitations of this retrospective pilot case series, lateral ridge augmentation using a collagenated porcine-derived xenograft combined with autogenous bone resulted in substantial horizontal bone gain and enabled successful implant-supported rehabilitation in the treated cases.

Histological and histomorphometric analyses demonstrated active bone formation, vascularization, and ongoing biomaterial-associated remodeling, supported by the presence of newly formed trabecular bone and TRAP-positive multinucleated giant cells at the graft interface. These findings suggest that the biomaterial may actively participate in the remodeling process under the evaluated clinical conditions.

Long-term follow-up demonstrated stable peri-implant conditions, maintained ridge dimensions, and absence of major biological or mechanical complications in this limited cohort.

Although constrained by a small sample size, a retrospective design, and the absence of a control group, these preliminary findings suggest that collagenated porcine-derived xenografts combined with autogenous bone may represent a clinically viable option for lateral ridge augmentation in severe horizontal ridge deficiencies. Further prospective controlled studies with larger patient populations are required to validate these observations and better define long-term predictability.

## Figures and Tables

**Table 1:** Clinical timeline according to CARE guidelines.

Time Point	Clinical Stage	Procedures/Findings
Baseline	Initial presentation	Severe horizontal ridge deficiency (RBW ≤ 4 mm); CBCT evaluation
Surgery (T0)	LRA procedure	GBR with autogenous bone + porcine xenograft; membrane placement
1 week	Early follow-up	Uneventful healing; no infection or dehiscence
1–3 months	Healing phase	Soft tissue maturation; no complications
3–5 months	Second-stage surgery	Implant placement + biopsy harvesting
3 months post-implant	Soft tissue augmentation	Connective tissue grafting/vestibuloplasty to improve peri-implant soft tissue quality
6 months post-implant	Prosthetic phase	Implant uncovering and prosthetic rehabilitation
Long-term follow-up	Outcome	Stable implants; maintained ridge width; healthy peri-implant tissues (mean 54.2 months)

**Table 2:** Clinical and radiographic outcomes following lateral ridge augmentation with porcine-derived xenograft.

No.	Sex	Age	Date of Surgery	Complications During Surgery	Major Complications After Surgery	Histology (Months)	Implant Site *	Initial RBW (mm)	Final RBW (mm)	Bone Gain (mm)	Follow-Up at 10 April 2026 (Months) **
1.	F	62	28 May 2021	None	none	4	4.5., 4.6., 4.7.	2	10–14	8–14	58.5
2.	M	34	26 October 2021	None	none	5	2.2., 2.4., 2.5.	2	12	10	53.5
3.	F	34	20 January 2022	None	none	3	1.2., 1.5.	2–4	10–12	8	50.7
Mean value	-	43.33	-	-	-	4	-	2.33	11.67	9.67	54.2

* FDI tooth-numbering system, ** After GBR surgery, F female, M male.

**Table 3:** Histological assessment of bone biopsy specimens following lateral ridge augmentation using porcine-derived xenograft.

No.	Biopsy ID	Time of Histology (Months)	New Bone (%)	Residual Graft Material (%)	Autogenous Bone Material (%)	Connective Tissue (%)	Vascularization	TRAP Activity	Overall Interpretation
1.	C1221-4	4	~40	~30	-	~30	Present (mature vessels)	Mild (TRAP−)	Active regeneration
2.	C-0622-1	5	~20	~20	-	~60	Well-developed (mature vessels)	TRAP+	Early regeneration
3.	C-0622-2	3	10–15	10–30	0–15	40–60	Present (heterogeneous maturity)	TRAP+/−	Ongoing remodeling

TRAP tartrate-resistant acid phosphatase.

## Data Availability

The data presented in this study are available on request from the corresponding author.

## Abbreviations

The following abbreviations are used in this manuscript:

LRA	lateral ridge augmentation
GBR	guided bone regeneration
RBW	residual bone width
CBCT	cone-beam computed tomography
OPG	orthopantomography
ASA	American Society of Anesthesiologists
PGCL	polyglycolidecaprolactone
ROI	region of interest
CT	connective tissue
EDTA	ethylenediaminetetraacetic acid
H&E	hematoxylin and eosin
TRAP	tartrate-resistant acid phosphatase
BSM	bone substitute material
ABM	autogenous bone material
NB	newly formed bone
MNGC/MNGCs	multinucleated giant cell(s)
